# Relative roles of *ABCG5/ABCG8* in liver and intestine[S]

**DOI:** 10.1194/jlr.M054544

**Published:** 2015-02

**Authors:** Jin Wang, Matthew A. Mitsche, Dieter Lütjohann, Jonathan C. Cohen, Xiao-Song Xie, Helen H. Hobbs

**Affiliations:** * Department of Molecular Genetics, University of Texas Southwestern Medical Center, Dallas, TX 75390; § Department of Internal Medicine, University of Texas Southwestern Medical Center, Dallas, TX 75390; ** Howard Hughes Medical Institute, University of Texas Southwestern Medical Center, Dallas, TX 75390; † Department of Clinical Pharmacology, University of Bonn, D-53105 Bonn, Germany

**Keywords:** cholesterol/absorption, bile, sterols, transport

## Abstract

ABCG5 (G5) and ABCG8 (G8) form a sterol transporter that acts in liver and intestine to prevent accumulation of dietary sterols. Mutations in either G5 or G8 cause sitosterolemia, a recessive disorder characterized by sterol accumulation and premature coronary atherosclerosis. Hepatic G5G8 mediates cholesterol excretion into bile, but the function and relative importance of intestinal G5G8 has not been defined. To determine the role of intestinal G5G8, we developed liver-specific (*L-G5G8^−/−^*), intestine-specific (*I-G5G8^−/−^*), and total (*G5G8^−/−^*) KO mice. Tissue levels of sitosterol, the most abundant plant sterol, were >90-fold higher in *G5G8^−/−^* mice than in WT animals. Expression of G5G8 only in intestine or only in liver decreased tissue sterol levels by 90% when compared with *G5G8^−/−^* animals. Biliary sterol secretion was reduced in *L-G5G8^−/−^* and *G5G8^−/−^* mice, but not in *I-G5G8^−/−^* mice. Conversely, absorption of plant sterols was increased in *I-G5G8^−/−^* and *G5G8^−/−^* mice, but not in *L-G5G8^−/−^* mice. Reverse cholesterol transport, as assessed from the fraction of intravenously administered ^3^H-cholesterol that appeared in feces, was reduced in *G5G8^−/−^*, *I-G5G8^−/−^*, and *L-G5G8^−/−^* mice. Thus, G5G8 expression in both the liver and intestine protects animals from sterol accumulation, and intestinal G5G8 contributes to extrahepatic cholesterol efflux in mice.

ABCG5 (G5) and ABCG8 (G8) play key roles in the maintenance of sterol balance in vertebrates. Inactivation of either protein causes sitosterolemia, an autosomal recessive disorder characterized by hypercholesterolemia, phytosterolemia, and premature coronary heart disease (1–3). Genetic ablation of G5G8 in mice has also been associated with disrupted cholesterol homeostasis (4), infertility and lipoatrophy (5), and liver abnormalities and cardiac lesions (6). G5 and G8 are expressed at significant levels in only two cell types: hepatocytes and enterocytes (2). The two proteins form heterodimers in the endoplasmic reticulum and then traffic to the apical membrane where they transport neutral sterols into bile or into the gut lumen (7). Phytosterols, which differ from cholesterol by modifications of the side chain, are the preferred substrates of the transporter (8).

Individuals with sitosterolemia have greatly reduced biliary sterol excretion (9, 10) as do mice in which either or both G5 or G8 have been inactivated (11–16). Expression of recombinant G5 and G8 in the liver can rescue the biliary sterol secretion defect in the *G5G8^−/−^* mice (17). The rate of excretion of biliary cholesterol is directly related to the level of hepatic G5G8 expression (17). These experiments have firmly established that hepatic G5G8 transports neutral sterols into bile.

The role of G5G8 in the intestine has been more difficult to define. Humans with sitosterolemia have increased fractional absorption of dietary cholesterol and plant sterols (10). *G5G8^−/−^* mice also have an increased fractional absorption of dietary phytosterols, but not of cholesterol (11). Based on these observations, it has been proposed that intestinal G5G8 exports dietary sterols into the gut lumen after they are transported into enterocytes via the sterol transporter Niemann-Pick C1-like 1 (NPC1L1) (18). However, direct support for this hypothesis is lacking. Biliary sterols compete with dietary sterols for uptake in the gut (19). It is possible that the increased fractional absorption of dietary phytosterols in *G5G8^−/−^* mice is a consequence of reduced biliary cholesterol levels due to absence of hepatic G5G8.

Several experimental models have been used to determine the relative roles of the intestine and liver in sterol excretion. Biliary secretion of cholesterol also requires ABCB4, which transports phospholipids into bile (13, 20). *Abcb4^−/−^* mice have levels of biliary cholesterol as low as those seen in *G5G8^−/−^* mice (13). Surprisingly, fecal sterol excretion is not reduced in the *Abcb4^−/−^* mice (13, 21). These results are not consistent with the premise that the biliary system is the major source of fecal sterols and thus the major pathway for neutral sterol excretion (22, 23).

Evidence from several sources has implicated the intestine as a major conduit for sterol excretion in mice (24, 25). This process, which has been referred to as transintestinal cholesterol excretion (26), requires the transit of sterols from the circulation through enterocytes into the gut lumen. Intestinal G5G8 may play a role in excretion of endogenous cholesterol rather than, or in addition to, dietary cholesterol (27).

A recent case report provided the first insights into the relative roles of intestinal and hepatic G5G8. Liver transplantation in a sitosterolemic individual with cirrhosis reduced his plasma plant sterols to near normal levels (28). Because the transplant presumably restored G5G8 activity in liver, but not intestine, the authors concluded that “the liver functions as the predominant organ for maintaining noncholesterol sterol balance.” Although the study was limited to a single case and the patient had severe liver disease of unknown etiology, these observations raise fundamental questions regarding the functional significance of intestinal G5G8. To determine the relative roles of hepatic and intestinal G5G8, we established mice in which G5G8 was inactivated selectively in the liver (*L-G5G8^−/−^* mice) or in the intestine *(I-G5G8^−/−^* mice). Sterol trafficking and regulation in these two new mouse models was compared with that observed in WT mice and mice expressing no G5G8 (*G5G8^−/−^* mice).

## MATERIALS AND METHODS

### Chemicals

Deuterated sterols were purchased from Medical Isotopes Inc. Radiolabeled sterols, including [^3^H]cholesterol, [^3^H]cholesteryl oleate, and [^14^C]cholesterol, were purchased from PerkinElmer Inc. Bile acid levels and phospholipids were measured using kits from Crystal Chem Inc. and Wako Chemicals USA Inc., respectively. Abs against mouse G5 and G8 were developed as previously described (11); Abs against ABCA1 were purchased from Pierce Biotechnology Inc. All other Abs, including polyclonal Abs against low density lipoprotein receptor (LDLR) and 3-hydroxy-3-methylglutaryl-CoA reductase (HMGCR) and monoclonal Abs against sterol-regulatory element binding protein (SREBP) 1 and SREBP-2 were gifts from Guosheng Liang (University of Texas Southwestern Medical Center).

### Generation of tissue-specific *G5G8^−/−^* mice

The *Cre-lox* strategy was used to inactivate G5 and G8 in either liver or intestine. *LoxP* sites were introduced into intron 2 of *Abcg5* (*G5*) and intron 1 of *Abcg8* (*G8*) using the oligonucleotide primers listed in supplementary Table 1. The construct was sequenced to ensure the integrity of the *loxP* sites prior to being transduced into C57Bl/6J mouse embryonic stem cells by electroporation. Homologous recombination occurred in 7 out of 352 clones and resulted in insertion of a *loxP* site and the *Frt*-flanked *Neo* cassette in intron 2 of *Abcg5* and a *LoxP* site in intron 1 of *Abcg8* (supplementary Fig. 1A). Both genes were inactivated subsequently by cre recombinase, which removed the first two exons of *G5* and the first exon of *G8* (supplementary Fig. 1A).

The map of the targeted allele was confirmed by genomic blotting, using *Nco*I- and *Scal*I/*Mef*I-digested genomic DNA from the clones (supplementary Fig. 1B). Probe 1 was generated by amplifying a 275 bp fragment from intron 2 of *G5*, and probe 2 was generated by amplifying a 250 bp DNA fragment from intron 3 of *G8* using the oligonucleotide primers provided in supplementary Table 1.

Three recombinant clones were injected into blastocysts of C57BL/6J mice. On day 2.5, the blastocysts were transferred into pseudopregnant recipient mice. A total of 17 (out of 72) of the offspring were chimeric (30–100%). Eleven mice transmitted the mutant allele to their offspring. Male offspring were crossed to female C57BL/6J mice. Offspring were screened by PCR (see supplementary Table 1 for oligonucleotides) and by genomic blotting. The F_1_ heterozygous mice were crossed to produce homozygotes (F_2_). The *Neo* cassette was removed by crossing homozygous mice to B6.Cg-Tg(ACTFLPe)9205Dym/J mice (obtained from Jackson Laboratory). The removal of the *Neo* gene in the offspring was confirmed by PCR using the primers listed in supplementary Table 1. Mice that were homozygous for alleles containing no *Neo* gene were crossed to B6.Cg-Tg(Alb-Cre)21Mgn/J, B6.SJL-Tg(Vil-cre)997Gum/J, and B6.Cg-Tg(CAG-cre/Esr1)5Amc/J (obtained from Jackson Laboratory) to generate liver-specific, intestine-specific, or total G5G8 KO mice, respectively. The genotypes of the mice were confirmed by PCR using the primers provided in supplementary Table 1.

### Animals and diets

Mice were housed in a temperature-controlled room (22°C) with a 12 h light cycle (6 AM to 6 PM) and 12 h dark cycle (6 PM to 6 AM). Mice were fed ad libitum a cereal-based rodent chow containing 4% fat and 115 μg/g cholesterol, 198 μg/g sitosterol, 63 μg/g campesterol, and 16 μg/g stigmasterol (Diet 7001; Harlan Teklad, Madison, WI). The mice were kept in wire-bottom cages to collect feces in some experiments.

All animal procedures were approved by the Institutional Animal Care and Research Advisory Committee at the University of Texas Southwestern Medical Center. At the end of each experiment, mice were euthanized with halothane followed by cervical dislocation prior to collecting tissues, which were all stored in liquid N_2_.

### Isolation of enterocytes

The small intestine was isolated and cut into three equal pieces, corresponding approximately to the duodenum, jejunum, and ileum. Each section was cut open lengthwise and rinsed thoroughly with PBS. Enterocytes were isolated from intestinal sections as described (29). In brief, intestinal sections were incubated in 15 ml of Krebs-Ringer bicarbonate buffer (120 mM NaCl, 4.6 mM KCl, 0.5 mM MgCl_2_, 10 mM d-glucose, 0.7 mM Na_2_HPO_4_, 1.5 mM NaH_2_PO_4_, 15 mM NaHCO_3_, 1 mM DTT, and 1.5 mM EDTA, pH 7.4) at 37°C in a shaking water bath for 20 min. The material was then passed through a 100 µM strainer. Cells were pelleted by centrifugation at 2,000 × *g* for 10 min before being frozen in liquid N_2_.

### mRNA expression profiling

RNA was prepared from mouse tissues using a total RNA isolation kit (Aurum Total RNA Fatty and Fibrous Tissue Kit, Bio-Rad). Quantitative real-time PCR was performed using total RNA isolated from each mouse sample (30). The oligonucleotides used for the RT-PCR assays are available upon request.

### Immunoblot analysis

Tissue membranes and nuclear fractions were prepared, and immunoblotting was performed as previously described (31).

### Sterol content of tissues

Sterol levels in plasma, gallbladder bile, and liver were measured by GC-MS. Plasma and tissues were saponified in 1 ml 90% ethanolic potassium hydroxide (1 N) at 100°C for 1 h after addition of 5α-cholestane and epicoprostanol as internal standards. Saponified samples were allowed to cool to room temperature, and 1 ml water was added to each tube. Lipids were extracted twice using 2 ml petroleum ether, and the organic phase from each extract was removed and dried under nitrogen. The residual lipids were redissolved in TRI-SIL reagent (Agilent) and derivatized at 75°C for 15 min. The samples were fractionated by GC using a 30 m DS5MS column, and the eluted sterols were identified and quantified by electron ionization-MS operating in single-ion-monitoring mode. Each sterol was identified using a pattern of three ions, and quantified using ions with the following *m/z*: cholesterol, 458; campesterol, 382; sitosterol, 396; and stigmasterol, 484.

### Fractional absorption of dietary sterols

The fractional absorption of sterols was measured as described (11). Mice were individually housed for 7 days prior to the experiment in wire-bottom cages. Each mouse was gavaged with 100 µl of medium-chain oil (Nestle Health Science) containing deuterated cholesterol, campesterol, sitosterol, and sitostanol in the amounts reported in the Results and figure legends. Feces were collected for 3 days, and the sterols were extracted as described (32). Fecal sterols and stanols were separated by GC, and the fractional absorption of sterols was calculated from the ratio of deuterated sterols over the “nonabsorbable” sterol, D_4_-sitostanol (10).

### Cholesterol transport assay

A total of 50–60 µCi of ^3^H-labeled cholesterol was dried under a stream of N_2_ and dissolved in 6 ml of 20% Intralipid (Baxter Healthcare). The lipid mixture was sonicated to incorporate the radioactive probe into the lipid micelles. Mice were housed in wire-bottom cages to facilitate stool collection. Each mouse received 0.2 ml of the Intralipid containing 40 mg of triglyceride and 1.7 µCi to 2 µCi of ^3^H-labeled cholesterol (13 ng of cholesterol) via tail vein injection. Blood was sampled 20 min after the injection. Feces were collected every 24 h for 3 days. At the end of 3 days, the mice were euthanized after a 4 h fast. Samples of gallbladder bile, plasma, and tissues were collected, and the sterols were extracted from the samples and the amount of radioactivity measured. Bile acids were extracted from the bile and feces as described (33).

### Statistical methods

Mean values were compared among groups using unpaired *t*-tests except for mRNA levels, which were compared using unpaired Mann-Whitney tests. All tests were performed using the GraphPad Prism Version 6 software package.

## RESULTS

### Generation of mice with intestine- and liver-specific inactivation of *G5* and *G8*

The Cre-lox system was used to generate tissue-specific *G5G8* KO mice (supplementary Fig. 1A). The targeting construct contained *loxP* sites in intron 2 of *G5* and intron 1 of *G8.* Homologous recombination between the targeting vector and the endogenous G5G8 gene generated an allele that could be inactivated in a tissue-selective fashion (supplementary Fig. 1A). PCR and genomic blot analyses were performed to confirm the restriction map of the targeted allele (supplementary Fig. 1B, C).

*G5G8* expression was reduced in tissues of mice containing the targeted allele that retained the *Neo* gene (data not shown). Therefore, the *Neo* gene was removed by crossing the mice with animals expressing *flp* recombinase under the control of the human actin β (ACTB) promoter (Jackson Laboratory). Successful removal of the *Neo* gene was confirmed by PCR amplification using oligonucleotides that flank the *neo* gene (supplementary Table 1 and supplementary Fig. 1).

**Fig. 1.**
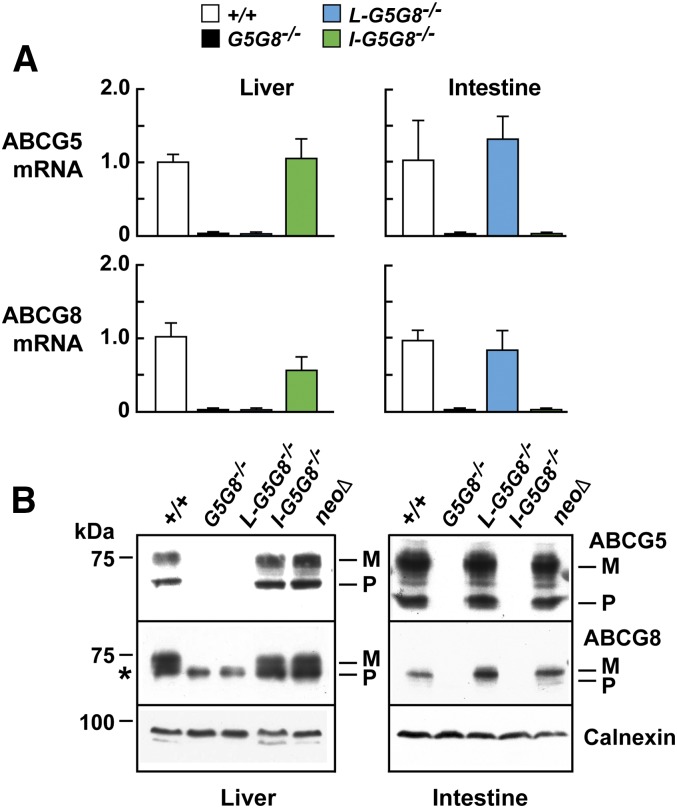
Expression of G5 and G8 mRNA (A) and protein (B) in the liver and intestine of WT, liver-specific (*L-G5G8^−/−^*), intestine-specific (*I-G5G8^−/−^*), and total *G5G8^−/−^* KO mice. A: RT-PCR was performed to quantitate levels of G5 and G8 mRNA in mice of the designated genotypes (five female mice/group, 12–15 weeks old). Cyclophilin was used as an internal control for mRNA expression. The mean values of the WT mice were set to 1. Standardized means ± SEM are shown. B: Immunoblot analysis of G5 and G8 in membranes isolated from the liver and intestine of mice was performed as described in the Materials and Methods. M, mature; P, precursor. The asterisk indicates a nonspecific band.

To ensure that both *G5* and *G8* were inactivated in a tissue-specific manner, we measured levels of G5 and G8 mRNA (**Fig. 1A**) and protein (Fig. 1B) in livers and intestines of the mice. No G5 or G8 mRNA or protein was detected in the livers of the *L-G5G8^−/−^* mice or in the intestine of *I-G5G8^−/−^* mice. Selective inactivation of G5G8 in one tissue did not alter the level of expression in the other tissue. The levels of G5G8 mRNA and protein in the intestines of the *L-G5G8^−/−^* mice and in the livers of the *I-G5G8^−/−^* were similar to the levels in WT animals.

Immunoblot analysis resolved bands corresponding to the precursor and the mature, glycosylated forms of G5 (Fig. 1B), which contains two N-linked sugars, but did not resolve the precursor and mature forms of G8, which only contains a single N-linked sugar (34). In the liver samples, a nonspecific band of ∼70 kDa was present in all four groups, including the *G5G8^−/−^* mice.

G5 and G8 mRNA and protein were expressed at similar levels in the *NeoΔ/NeoΔ* and WT mice; therefore, the *NeoΔ/NeoΔ* mice were used as controls in the remainder of the experiments.

### *G5G8* expression in either the liver or intestine is sufficient to reduce plant sterol levels in tissues

**Fig. 2.**
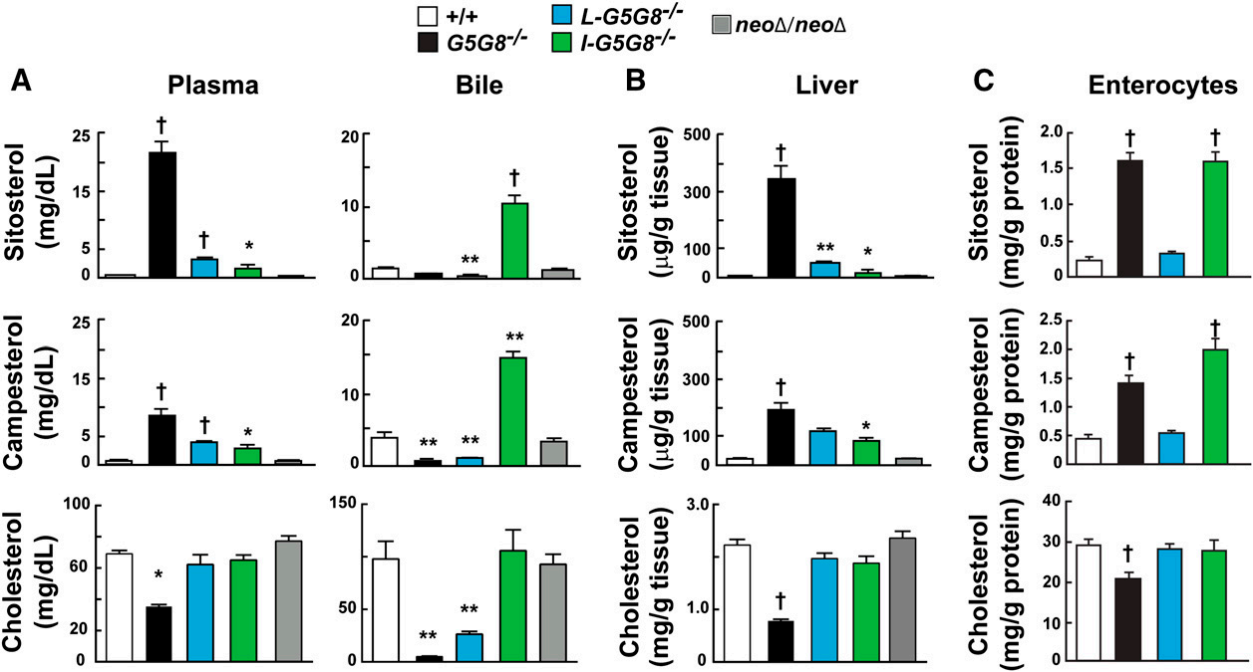
Sterol levels in the plasma and gallbladder bile (A), liver (B), and enterocytes (C) of tissue-specific *G5G8^−/−^* mice. Tissues and cells were collected as described in the Materials and Methods. Mice in which the *Neo* cassette had been removed were used as controls (*NeoΔ*/*NeoΔ*). Female mice (n = 5/group, 10–12 weeks old) were fed a chow (Diet 7001) for 6 weeks before being euthanized. Tissue sterols were analyzed using GC-MS as described the Materials and Methods. This experiment was repeated three times, and the results were similar. Values are means ± SEMs. All statistical comparisons are to the WT animals. * *P* < 0.05, ** *P* < 0.01, and ^†^ *P* < 0.001.

Plasma levels of cholesterol and the two most abundant dietary plant sterols, sitosterol and campesterol, were measured in plasma, gallbladder bile, livers, and enterocytes of the four strains of mice (**Fig. 2**). The mean plasma levels of sitosterol and campesterol were increased 95-fold and 14-fold in in the *G5G8^−/−^* mice compared with WT littermates (Fig. 2A). Plasma plant sterol levels were significantly lower in *I-G5G8^−/−^* and *L-G5G8^−/−^* mice than in *G5G8^−/−^* mice, but higher than in the WT mice. Plant sterol levels were reduced by similar amounts in the two strains of tissue-specific KO mice. Thus, G5G8 expression in either the intestine or liver is sufficient to dramatically lower plasma phytosterol levels relative to *G5G8^−/−^* animals, but G5G8 expression in both tissues is required to reduce levels to those seen in WT animals.

Phytosterol levels in WT mice were similar to those in *NeoΔ/Neo*Δ mice, as expected based on the expression levels of the G5 and G8 mRNA and protein (Fig. 1).

*G5G8^−/−^* mice had significantly lower circulating levels of cholesterol than did WT animals, as reported previously (11). Plasma cholesterol levels did not differ significantly between the *L-G5G8^−/−^*,* I-G5G8^−/−^*, and WT mice.

Levels of sitosterol and campesterol were low in gallbladder bile of both the total *G5G8^−/−^* and *L-G5G8^−/−^* mice (Fig. 2A, right). In contrast to the *L-G5G8^−/−^* mice, inactivation of *G5G8* selectively in the intestine resulted in a striking increase in biliary plant sterol levels, presumably due to increased intestinal absorption of the dietary phytosterols.

The level of biliary cholesterol was higher in the *L-G5G8^−/−^* mice than in the total KO animals (5 ± 0.4 mg/dl vs. 26 ± 2.7 mg/dl, *P* = 0.001). To determine whether the increase in cholesterol content of the bile in the *L-G5G8^−/−^* mice is due to cholesterol secreted from the gallbladder, where G5 and G8 have been shown previously to be expressed (35), we measured the mRNA levels of G5 and G8 in these mice and compared them with the WT and total KO mice (supplementary Fig. 2). The levels of mRNA for G5 and G8 in the gallbladders of *L-G5G8^−/−^* mice were as low as those of the *G5G8^−/−^* animals. Thus, the small amount of cholesterol that is present in the bile of the *L-G5G8^−/−^* is not due to the effect of G5G8 expression in the gallbladder. The higher levels of biliary cholesterol in the liver-specific KO mice may be due to an increase in the rate of hepatic cholesterol synthesis in the *L-G5G8^−/−^* mice. Hepatic levels of the plant sterol sterol stigmasterol, which suppresses SREBP activation (36), were ∼6-fold lower in the *L-G5G8^−/−^* mice than the *G5G8^−/−^* mice (0.7 ± 1 vs. 4.4 ± 0.4 μg/g, *P* < 6 × 10^−6^).

Sterol levels were also measured in duodenal enterocytes from these mice (Fig. 2C). The levels of phytosterols were substantially increased in enterocytes from the *I-G5G8^−/−^* mice and the *G5G8^−/−^* mice, but not from the *L-G5G8^−/−^* mice. The levels of cholesterol in the *G5G8^−/−^* mice were significantly lower than in the other three strains of mice; it is unclear why the cholesterol levels are not reduced to the same extent in the *I-G5G8^−/−^* mice, given the very high levels of plant sterols in the enterocytes of these animals (Fig. 2C).

Thus, expression of *G5G8* in either the liver or intestine is sufficient to markedly reduce plant sterol levels in tissues compared with *G5G8^−/−^* mice, though not to the very low levels seen in WT animals. Expression of G5G8 in both the liver and the intestine contribute importantly to sterol homeostasis.

### Increased absorption of phytosterols but not cholesterol in G5G8 KO mice

**Fig. 3.**
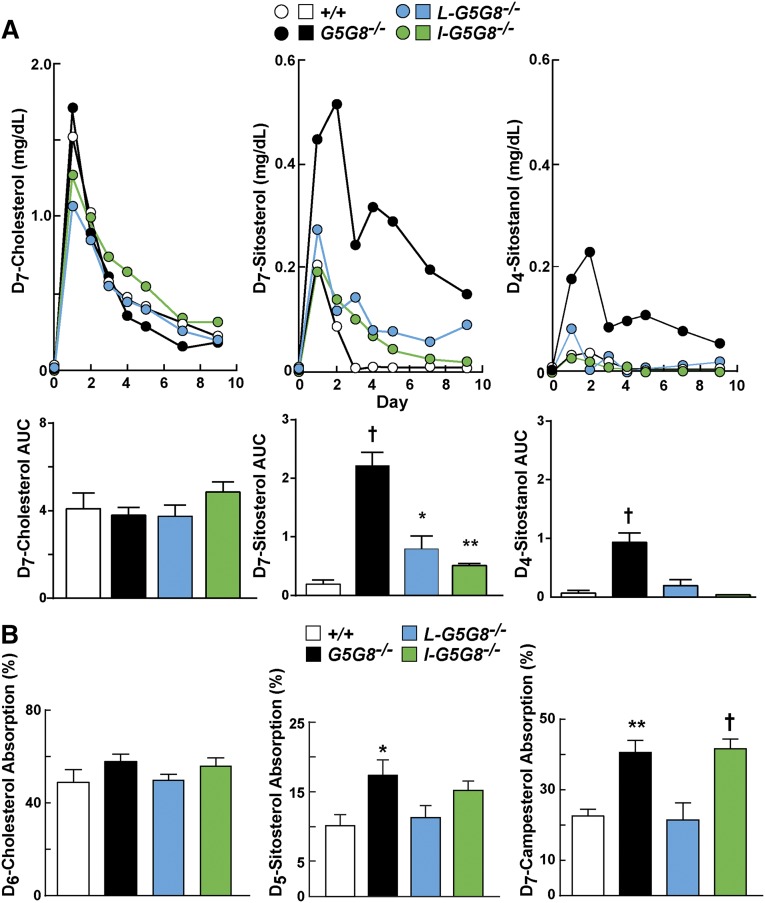
Appearance of deuterated dietary sterols in plasma (A) and fractional sterol absorption (B) in tissue-specific *G5G8^−/−^* mice. A: Male mice (n = 6/group, 10–13 weeks old) were individually housed for 7 days. Venous blood was sampled from the tail vein prior to the mice being gavaged with medium-chain triglycerides (100 µl) containing D_7_-cholesterol (200 µg), D_7_-sitosterol (100 µg), and D_4_-sitostanol (100 µg). Blood samples were collected daily for 9 days, and the sterols were extracted, separated by GC, and quantitated, as described in the Materials and Methods. The AUCs of the plasma sterol levels are provided. B: Fractional absorption of sterols. Female mice (n = 5/group, 23–25 weeks old) were individually housed in wire-bottom cages, and the fractional absorption of the indicated sterols was performed using D_6_-cholesterol, D_5_-sitosterol, and D_7_-campesterol. Feces were collected for 3 days prior to and after being gavaged with the labeled sterols. Sterols were processed and measured as described in A. Values are means ± SEMs.

Next, we measured and compared the absorption of dietary sterols among the four strains of mice. Mice were given an oral bolus of labeled cholesterol, sitosterol, and a nonabsorbed sterol, sitostanol. Blood samples were collected at the indicated times over 10 days, and the levels of the deuterated sterols were measured (**Fig. 3A**). No significant differences were seen in the appearance or clearance of D_7_-cholesterol from the circulation among the mice. In contrast to these results, the plasma levels of D_7_-sitosterol were dramatically increased in the *G5G8^−/−^* mice. No differences in the plasma levels of D_7_-sitosterol were seen at the initial time points in either tissue-specific KO strain, but the rate of clearance of sitosterol from the circulation was reduced in both strains of tissue-specific KO mice when compared with WT littermates.

### Sitostanol is a substrate of G5G8

As expected, only a very small fraction of the orally administered D_4_-sitostanol was detected in the blood of the WT mice, even at the earliest time point (Fig. 3A, upper right panel). A similar, barely detectable increase in D_4_-sitostanol was detected in the circulation of the *I-G5G8^−/−^* mice. The level of D_4_-sitostanol was slightly higher at the first time point in the *L-G5G8^−/−^* mice. We also quantified the area under the curve (AUC) over the 10 day period (Fig. 3A, bottom). The AUC in the two tissue-specific KO mouse strains was not significantly different from the WT animals (Fig. 3A, lower right panel). In contrast to these results, the D_4_-sitostanol levels in the plasma were higher over the entire course of the experiment in the *G5G8^−/−^* mice, and the AUC was significantly higher in the *G5G8^−/−^* mice than in the WT animals. These results are consistent with the notion that sitostanol is a substrate for G5G8 and accumulates in tissues in its absence.

### Increased fractional absorption of phytosterols, but not cholesterol, in intestine-specific *G5G8^-^*^/-^ mice

The measurement of fractional absorption of dietary sterols is determined using a nonabsorbed sterol, typically sitostanol, which serves as a recovery standard. Because sitostanol is a substrate of G5G8 (Fig. 3, right panel), the fractional absorption of sterols underestimates the actual values in *G5G8^−/−^* mice. Given that caveat, no significant differences were found between the strains in the fractional absorption of cholesterol (Fig. 3B, left panel). The fractional absorption of sitosterol and campesterol was increased in the *G5G8^−/−^* mice and *I-G5G8^−/−^* mice compared with the other two groups of mice, although the difference was not significant for sitosterol in the *I-G5G8^−/−^* mice (Fig. 3B, middle and right panel).

### Altered mRNA and protein expression in small intestine of *G5G8^−/−^* mice

**Fig. 4.**
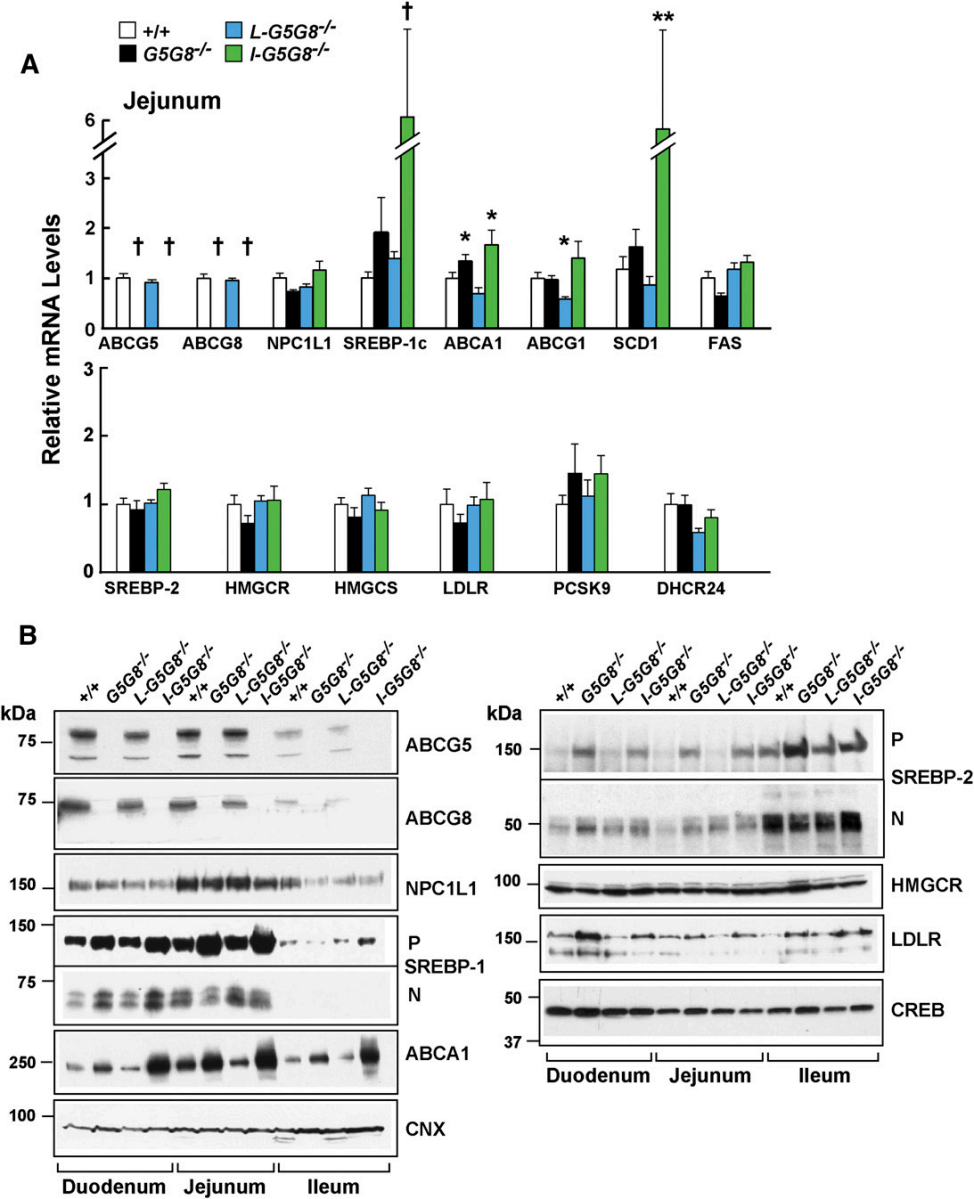
Levels of selected mRNAs (A) in the jejunum (enterocytes) and proteins in the intestine (B) of WT, *G5G8^−/−^*, *L-G5G8^−/−^*, and *I-G5G8^−/−^* female mice (n = 5/group, 23–25 weeks old). Total RNA was isolated from the jejunum of each mouse and the relative mRNA levels were measured using quantitative real-time PCR as described in the Materials and Methods. Cyclophilin was used as an internal control, and the level was expressed relative to the level of the transcript in the WT animals, which was set to 1. Values are means ± SEMs. B: Enterocytes were isolated from the three sections of intestine as described in the Materials and Methods. Membrane and nuclear fractions were prepared as described in the Materials and Methods. A total of 35 μg of protein was subjected to SDS-PAGE and immunoblotted with Abs to the indicated proteins as described in the Materials and Methods. This experiment was repeated three times, and the results were similar. CNX, calnexin; CREB, cAMP response element binding protein; DHCR24, desmosterol reductase; FPPS, farnesyl pyrophosphate synthase; SCD1, stearolyl-CoA desaturase-1; SS, squalene synthase.

Next, we examined the effect of tissue-specific inactivation of G5G8 on expression of selected mRNA transcripts and proteins involved in lipid metabolism and transport (**Fig. 4**). Expression of the sterol transporter NPC1L1 was higher in the jejunum than in either the duodenum or ileum, as has been reported previously (37), but no differences in the levels of NPC1L1 mRNA (Fig. 4A) or protein (Fig. 4B) were observed among the four strains of mice.

mRNA levels of several liver X receptor (LXR) target genes, including *SREBP-1c*, *ABCA1*, and *SCD1*, were increased in the intestines of *I-G5G8^−/−^* mice, although no significant increases were seen in FAS mRNA levels (Fig. 4A). The increase in SREBP-1c mRNA was associated with an increased level of the active, nuclear form of the transcription factor in duodenal enterocytes from both the total KO and the *I-G5G8^−/−^* mice. The changes were more modest in the jejunum. The increase in ABCA1 mRNA was associated with an increase in protein in all three segments of the intestine in the total KO and *I-G5G8^−/−^* mice (Fig. 4B).

SREBP-2 transcript levels were increased in the jejuna of the *G5G8^−/−^* mice. The increase in SREBP-2 mRNA was associated with an increase in both the precursor and nuclear form of SREBP-2 protein in the duodenum. In the jejunum, the precursor form of SREBP-2 was higher in the *G5G8^−/−^* and the *I-G5G8^−/−^* mice (Fig. 4B), although the mature form of the transcription factor was higher in all the KO mice when compared with WT animals.

The levels of SREBP-2 target genes encoding enzymes in the cholesterol biosynthetic pathway, namely HMGCR, 3-hydroxy-3-methylglutaryl-CoA synthase (HMGCS), and DHCR24, were not significantly different in the three genetically modified lines of mice from those of WT mice. Although the levels of LDLR mRNA did not differ among the four strains (Fig. 4A), the levels of LDLR protein were increased in the duodenum and jejunum of both the *G5G8^−/−^* and *I-G5G8^−/−^* mice. The levels of another SREBP-2 target gene, proprotein convertase subtilisin/kexin type 9 (*PCSK9*), were increased, though not significantly, in the *G5G8^−/−^* and the *I-G5G8^−/−^* mice.

**Fig. 5.**
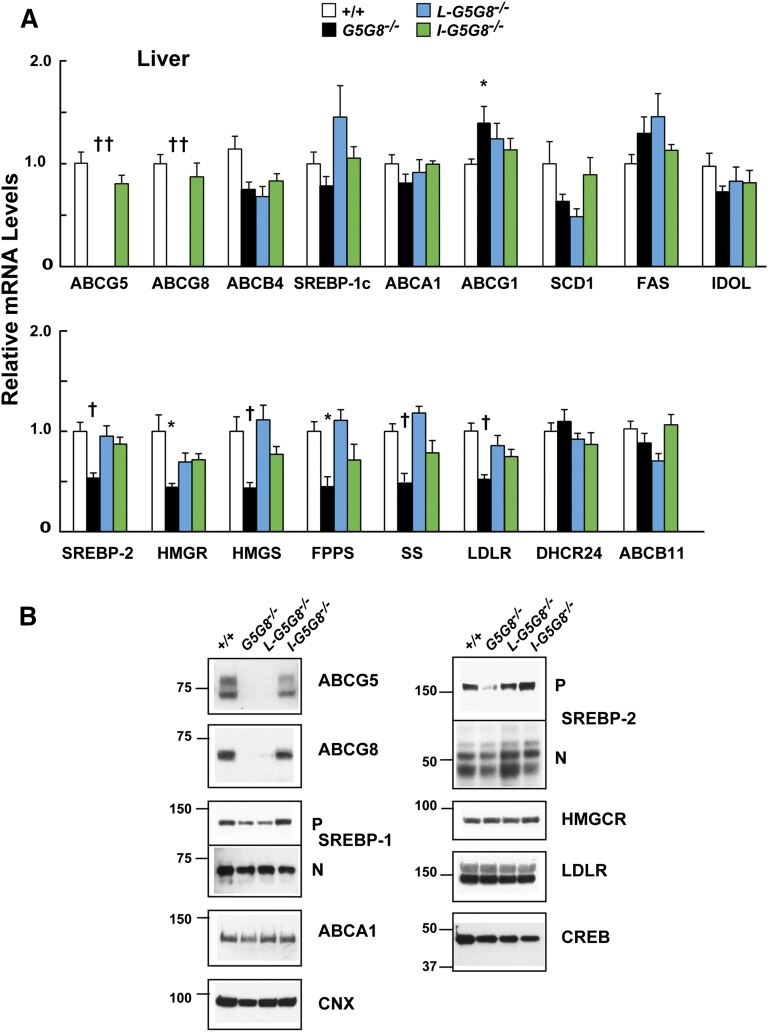
Levels of selected mRNAs (A) and proteins (B) in the livers of WT, *G5G8^−/−^*, *L-G5G8^−/−^*, and *I-G5G8^−/−^*female mice (n = 5/group, 23–25 weeks old). Total RNA was isolated from the liver of each mouse, and the relative mRNA levels were measured using quantitative real-time PCR as described in the Materials and Methods. Cyclophilin was used as an internal control, and the level was expressed relative to the level of the transcript in the WT animals, which was set to 1. Values are means ± SEMs. B: Membrane and nuclear fractions were prepared as described in the Materials and Methods. A total of 35 μg of hepatic protein was subjected to SDS-PAGE and immunoblotted with Abs to the indicated proteins as described in the Materials and Methods. This experiment was repeated twice, and the results were similar.

A similar analysis was performed in the livers of the four groups of mice (**Fig. 5A**). No significant changes were found in the levels of SREBP-1c mRNA or protein among the mouse strains (Fig. 5B). Nor were any consistent changes apparent in the LXR and SREBP-1c target genes. MRNA levels of one LXR target, ABCG1, were significantly increased in the livers of the *G5G8^−/−^* mice.

Levels of SREBP-2 mRNA were reduced by ∼40% in the livers of the *G5G8^−/−^* mice. This reduction was associated with a reduction in mRNA levels of several SREBP-2 target genes, including *Hmgcr*, *Hmgcs*, *Fpps*, *Ss*, *Ldlr*, and *Pcsk9*. Despite the reduction in mRNA levels of these target genes, no differences were detected in the corresponding proteins encoded by a subset of these transcripts, including the LDLR and HMGCR.

### G5G8 in liver and intestine both contribute to cholesterol excretion

**Fig. 6.**
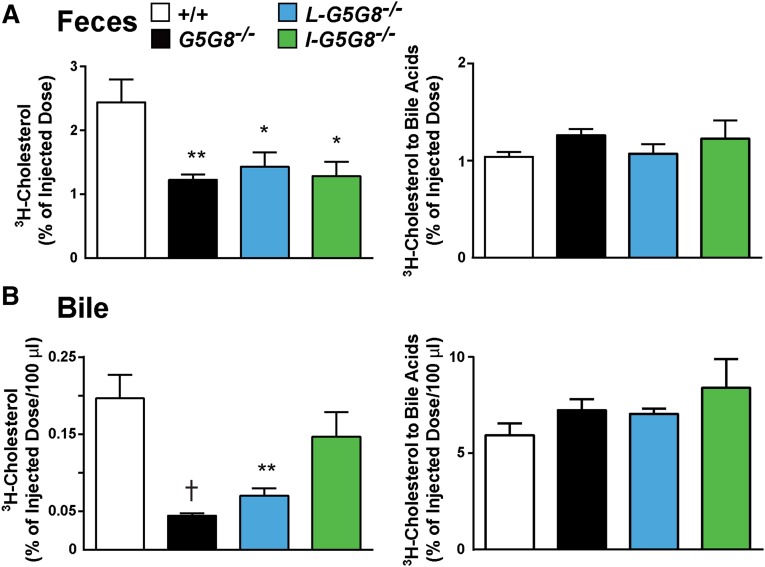
Contribution of G5G8 to fecal excretion of cholesterol. A total of 60 µCi of ^3^H-labeled cholesterol were dried and dissolved in 6 ml of 20% Intralipid (Baxter Healthcare). The lipid mixture was sonicated, and 0.2 ml was injected into the tail veins of six male mice/group (21–24 weeks old). Each mouse received 40 mg of triglyceride and 13 ng of cholesterol (2 μCi). The feces were collected for 3 days, and the fraction of the radiolabeled cholesterol that was injected and appeared as sterol or bile acid in the feces (A) or gallbladder bile (B) was quantitated. This experiment was repeated twice, and the results were similar. Values are means ± SEMs. * *P* < 0.05, ** *P* < 0.01, and ^†^ *P* < 0.001.

To determine whether intestinal G5G8 contributes to the excretion of sterols from extrahepatic tissues, we examined the clearance of ^3^H-cholesterol from the blood into the feces. Tritiated cholesterol was incorporated into Intralipid (200 μl) and injected into the tail veins of male mice (six per group). A significant decrease in the amount of ^3^H-cholesterol excreted into the feces was observed in *G5G8^−/−^* mice (**Fig. 6A**, left). A decrease in excreted ^3^H-cholesterol of a similar magnitude was seen in both of the tissue-specific KO strains. In contrast to these results, no differences were seen in the amount of cholesterol that was converted to bile acids and excreted in the feces (Fig. 6A, right). The plasma levels of ^3^H-cholesterol were measured 20 min after the injection of the sterol and at the end of the experiment (72 h) (supplementary Fig. 3). There was a slight reduction in the plasma level of ^3^H-cholesterol in the liver-specific KO mice at 20 min (*P* = 0.043) and no differences between the strains at 72 h (supplementary Fig. 2). Therefore, the reduced appearance of intravenously administered cholesterol in the feces of the three KO mouse strains is not due to differences in clearance from the circulation. Taken together with the data shown in Fig. 3, this finding suggests that G5G8 has little impact on the kinetics of cholesterol in plasma, but that expression of the transporter in both liver and intestine promotes excretion of cholesterol into the feces.

As expected, the amounts of ^3^H-cholesterol secreted into bile were lower in the *L-G5G8^−/−^* and *G5G8^−/−^* mice than in the WT or *I-G5G8^−/−^* animals (Fig. 6B, left).

### Fecal excretion of phytosterol and cholesterol were unchanged in *G5G8^−/−^* mice

**Fig. 7.**
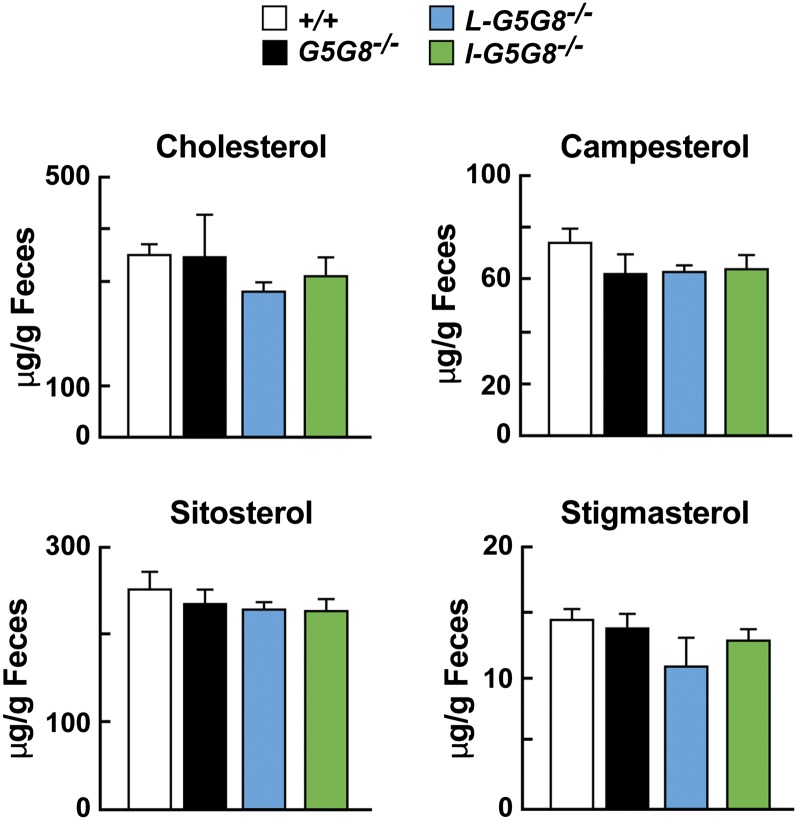
Total sterol excretion in *G5G8^−^*^/−^ mice. Mice were individually housed for 7 days prior to the experiment. Feces were collected from 12- to 14-week-old male mice (n = 5/group) for 3 days while they consumed a chow diet. Sterols were processed and measured as described in the Materials and Methods. The experiment was repeated three times, and the results were similar. Values are means ± SEMs.

Finally, we compared the daily mass of cholesterol, campesterol, sitosterol, and stigmasterol in the feces of the mice over a 3 day period. Despite major differences in the rates of sterol absorption in the different mouse strains (Fig. 3), no statistical differences were found in the total amount of cholesterol, campesterol, sitosterol, or stigmasterol among the strains (**Fig. 7**).

## DISCUSSION

The major finding of this paper is that expression of G5 and G8 in either the intestine or liver is sufficient to limit accumulation of plasma phytosterol levels, but expression in both tissues is required to maintain the very low levels observed in WT animals. Liver- and intestine-specific *G5G8^−/−^* mice have an intermediate phenotype (Fig. 2), with dietary phytosterol accumulation well beyond the range seen in normal mice, but much less than in the total *G5G8^−/−^* mice.

Mice expressing no G5 or G8 have increased fractional absorption of dietary plant sterols (11). It has been unclear whether the increase is caused by a failure to efflux plant sterols from enterocytes due to lack of intestinal G5G8 or by decreased competition for NPC1L1-mediated sterol uptake due to a decrease in hepatic G5G8-mediated delivery of biliary sterols to the gut lumen (19). Here we showed that inactivation of G5G8 in the intestine resulted in increases in biliary sterol secretion and in the fractional absorption of plant sterols, but not of dietary cholesterol (Figs. 2, 3B). Thus, the increase in fractional absorption of dietary plant sterols in the *G5G8^−/−^* mice is a direct result of the loss of G5G8 function in the enterocytes, not a secondary consequence of reduced sterol secretion into bile.

The finding that plant sterols accumulate to similarly high levels in the enterocytes of *I-G5G8^−/−^* and total *G5G8^−/−^* mice (Fig. 2) provides further evidence that intestinal G8G8 acts to limit plant sterol uptake by enterocytes. Plant sterols that evade G5G8 in the enterocytes are incorporated into chylomicrons and ultimately enter the liver with chylomicron remnants. Thus, the amounts of plant sterols entering the liver are greater in both *I-G5G8^−/−^* and total *G5G8^−/−^* mice than in WT or *L-G5G8^−/−^* animals. In *I-G5G8^−/−^* mice, the excess plant sterols are efficiently secreted into bile by hepatic G5G8 (Fig. 2). Consequently, biliary plant sterols are greatly increased in these animals compared with either WT or total *G5G8^−/−^* mice, whereas levels of plant sterols in the plasma and liver are only modestly increased. Conversely, in the total *G5G8^−/−^* mice the lack of hepatic G5G8 precludes effective biliary secretion of plant sterols. Accordingly, levels of plant sterols remain low in the bile but accumulate to high levels in the plasma and livers of these animals (Fig. 2).

Sitostanol is routinely used as a nonabsorbable sterol that serves as an internal control when measuring fractional absorption of dietary sterols (32, 38). In the WT mice in this study, the AUC of plasma sitostanol concentrations plotted against time was very low (Fig. 3), which is consistent with the notion that sitostanol is very poorly absorbed (39). However, in the intestine-specific and the total G5G8 KO animals, sitostanol absorption was significantly increased (Fig. 3), indicating that sitostanol is a substrate of G5G8. Wang et al. (40) also found a disproportionate increase in absorption of sitostanol relative to cholesterol in G8 KO mice. A relative increase in sitostanol absorption would lead to an underestimation of the true fractional sterol absorption. Thus, the true fractional sterol absorption in the *G5G8^−/−^* and *I-G5G8^−/−^* mice may be higher than the estimates obtained in our studies.

For logistical reasons, each individual experiment was performed in either male or female mice. In a previous study, we found that total deletion of G5G8 in mice had similar effects on sterol levels and metabolism (absorption, biliary excretion) in male and female animals (11). In the present study, separate experiments were performed in male and female mice to assess the effects of tissue-specific G5G8 deletion on tissue sterol levels, gene expression, and biliary cholesterol excretion (data not shown). None of these parameters showed systematic sex differences.

We showed previously that G5G8 shows strong selectivity among different neutral sterols, secreting sitosterol > campesterol > cholesterol (8). As a result, sitosterol, the most abundant sterol in the diet (mouse chow contains 198 μg/g sitosterol and 63 μg/g campesterol), is almost invariably present at lower concentrations than campesterol in the tissues and plasma of normal humans and WT mice. In contrast, in *G5G8^−/−^* mice the ratio of sitosterol to campesterol approaches that of the diet (8). The ratio of sitosterol to campesterol in plasma of the tissue-specific KO mice remained slightly, but significantly, higher than that of WT mice (2.2 in the total KO, 0.75 in the *L-G5G8^−/−^*, and 0.56 in the *I-G5G8^−/−^* mice vs. 0.25 in WT mice). Thus, expression of G5G8 in either liver or intestine confers a high degree of sterol selectivity, but expression at both sites is required to achieve the level of selective sterol excretion observed in WT animals.

The strong preference of G5G8 for noncholesterol sterols is essential to the maintenance of cholesterol homeostasis because even modest changes in sterol structure can profoundly alter interaction with the sterol regulatory machinery (36). Severe disruption of cholesterol homeostasis by noncholesterol sterols is illustrated by the marked depletion of cholesteryl esters in the adrenal glands of *G5G8^−/−^* mice (4). Despite a 90% reduction in cholesterol levels, cholesterol synthesis is suppressed and cholesterol excretion is upregulated in the adrenal glands of these animals (4). These effects result from inhibition of SREBP processing and activation of LXR, respectively, and appear to be mediated by sterols with unsaturated side chains (36).

Upregulation of LXR-responsive genes was readily apparent in enterocytes from *I-G5G8^−/−^* mice, in which the levels of SREBP-1c and ABCA1 mRNAs were 5.4-fold and 1.6-fold higher, respectively, than those of WT mice. These changes in mRNA levels were associated with increased levels of SREBP-1c and ABCA1 protein. Despite apparently similar increases in plant sterol levels, expression of LXR-targets was lower in jejunocytes from *G5G8^−/−^* mice. The reasons for this disparity are not known. In contrast to our findings in liver and our previous data from adrenal glands (4), levels of SREBP-2 protein were increased in duodenal and jejunal segments of *G5G8^−/−^* and *I-G5G8^−/−^* mice. These increases appeared to be posttranslational, as jejunal levels of SREBP-2 mRNA were not increased in these animals, and were not accompanied by increased expression of key SREBP-2 target genes such as *Hmgcr* and *Hmgcs*. Whereas these changes do not mirror those observed in liver and adrenal of sitosterolemic mice, the anomalous increase in intestinal SREBP-2 levels and the dissociation between the nuclear form of the transcription factor and expression of its target genes indicates that plant sterol accumulation disrupts the cholesterol homeostatic machinery in enterocytes.

MRNA levels of SREBP-2 and several of its target genes were decreased by ∼50% in livers of total KO animals, consistent with our previous findings (8). No significant changes in expression of sterol-regulatory genes were observed in livers of tissue-specific KO mice. Thus, expression of G5G8 in either liver or intestine is sufficient to protect the liver against the disruptive effects of noncholesterol sterols.

Biliary cholesterol levels in the *L-G5G8^−/−^* mice, although low, were about three times higher than in the *G5G8^−/−^* mice (Fig. 2A). When sterol absorption is blocked by ezetimibe (41), or by genetic disruption of NPC1L1 (42), biliary cholesterol in the *G5G8^−/−^* mice increases to levels that are comparable to those of *L-G5G8^−/−^* mice. These findings support the hypothesis that a portion of biliary cholesterol enters the bile via a G5G8-independent mechanism, and that the very low levels of biliary cholesterol in *G5G8^−/−^* mice are due in part to cholesterol depletion in the liver. The relative increase in biliary cholesterol levels of the *L-G5G8^−/−^* mice may reflect a higher rate of sterol synthesis secondary to lower plant sterol levels in the livers of these mice compared with the total KO *G5G8* mice.

All cells (except red blood cells) synthesize cholesterol to replenish their membranes and for the production of hormones and bile acids. Mice synthesize ∼160 mg cholesterol/kg body weight/day (43). To maintain cholesterol balance, an equivalent amount must be excreted, either in the form of bile acids or as free cholesterol. The major pathway for reverse cholesterol transport in mammals has been thought to be via the bile (43). The ∼75% reduction in fecal cholesterol excretion in patients with sitosterolemia (1, 44, 45) is consistent with this model. In contrast, fecal sterol excretion is normal, or only modestly reduced, in several mouse models in which biliary cholesterol secretion is disrupted (13, 22). These data have implicated the presence of nonbiliary reverse cholesterol transport pathway, in which sterols are transported from the blood across the enterocytes into the gut lumen (27, 46). The molecular basis of this pathway has not been defined. In the present study, fecal excretion of intravenously administered cholesterol was impaired in all three G5G8 KO strains, while excretion of bile acids derived from cholesterol was similar to those of WT animals. These findings suggest that neutral sterols enter the feces through both biliary and nonbiliary routes and that G5G8 contributes to both pathways. The transintestinal pathway would be predicted to predominate in mice but is unlikely to be a major pathway for cholesterol excretion in humans, given the very low fecal sterol excretion rates in humans with sitosterolemia.

## Supplementary Material

Supplemental Data
