# Supplementary material for: Relative roles of ABCG5/ABCG8 in liver and intestine
Source: J Lipid Res. 2015 Feb;56(2):319–30. doi: 10.1194/jlr.M054544 (PMC4306686; doi:10.1194/jlr.M054544)
Supplement: Supplemental Data [file supp_56_2_319__index.html]

Relative Roles of ABCG5/ABCG8 in Liver and Intestine — Relative roles of ABCG5/ABCG8 in liver and intestine — Supplemental Data 

# Relative roles of *ABCG5/ABCG8* in liver and intestine

## Supplemental Data

**Files in this Data Supplement:**

- Supplemental Materials - Map of the G5G8 locus and the targeting construct used to inactivate the gene by homologous recombination
