# Supplementary material for: Relative roles of ABCG5/ABCG8 in liver and intestine
Source: J Lipid Res. 2015 Feb;56(2):319–30. doi: 10.1194/jlr.M054544 (PMC4306686; doi:10.1194/jlr.M054544)
Supplement: Supplemental Data [file supp_M054544_jlr.M054544-1.pdf]

SUPPLEMENTAL MATERIALS

Fig. SI: Map of the G5G8 locus and the targeting construct used to inactivate the gene by homologous recombination. The *Cre-lox* approach was used to selectively inactivate *Abcg5* and *Abcg8* in either liver or intestine, as described in the methods. *LoxP* sites were inserted into intron 2 of *Abcg5* and intron 1 of *Abcg8* and the targeting construct was introduced into embryonic stem cells (ES). A: Map of the G5G8 locus, the targeting construct, the targeted allele, and the disrupted allele are shown. B: Ethidium-stained agarose gel of PCR amplified products from wild-type (734 bp), *floxed* (2613 bp), and *floxed*Δ*neo* (934 bp) alleles. C: Genomic blot analysis of *Nco*I (upper panel) and *Sca*I/*Mef*I (lower panel)-digested DNA from mice of the indicated genotypes.
DTA, diphtheria toxin A-fragment gene.

Fig. S2: Levels of selected mRNA in gallbladder of wild-type, *G5G8*$^{-/-}$, and *L-G5G8*$^{-/-}$ female mice (n=3/group, 23-25 week old). Total RNA was isolated from the gallbladders in each group, and the relative mRNA levels were measured using quantitative real-time PCR as described in the Methods. Cyclophilin was used as an internal control and the level was expressed relative to the level of the transcript in the wild-type animals, which was set to 1.

Fig. S3: Levels of tritiated cholesterol in the plasma of mice. A total of 13 ng of ^{3}H-cholesterol (2 μCi) in 40 mg of intralipid (0.2 ml) was injected into the tail vein of each mouse (6 male mice/group, 21-24 week old). Blood was sampled (25 μl) 20 min after injection and again after 72 hours and the radioactivity was quantified in a scintillation counter. This experiment was repeated twice and the results were similar. Values are Means±SEMs. *P<0.05, **P<0.01 and † P<0.001

Figure S1

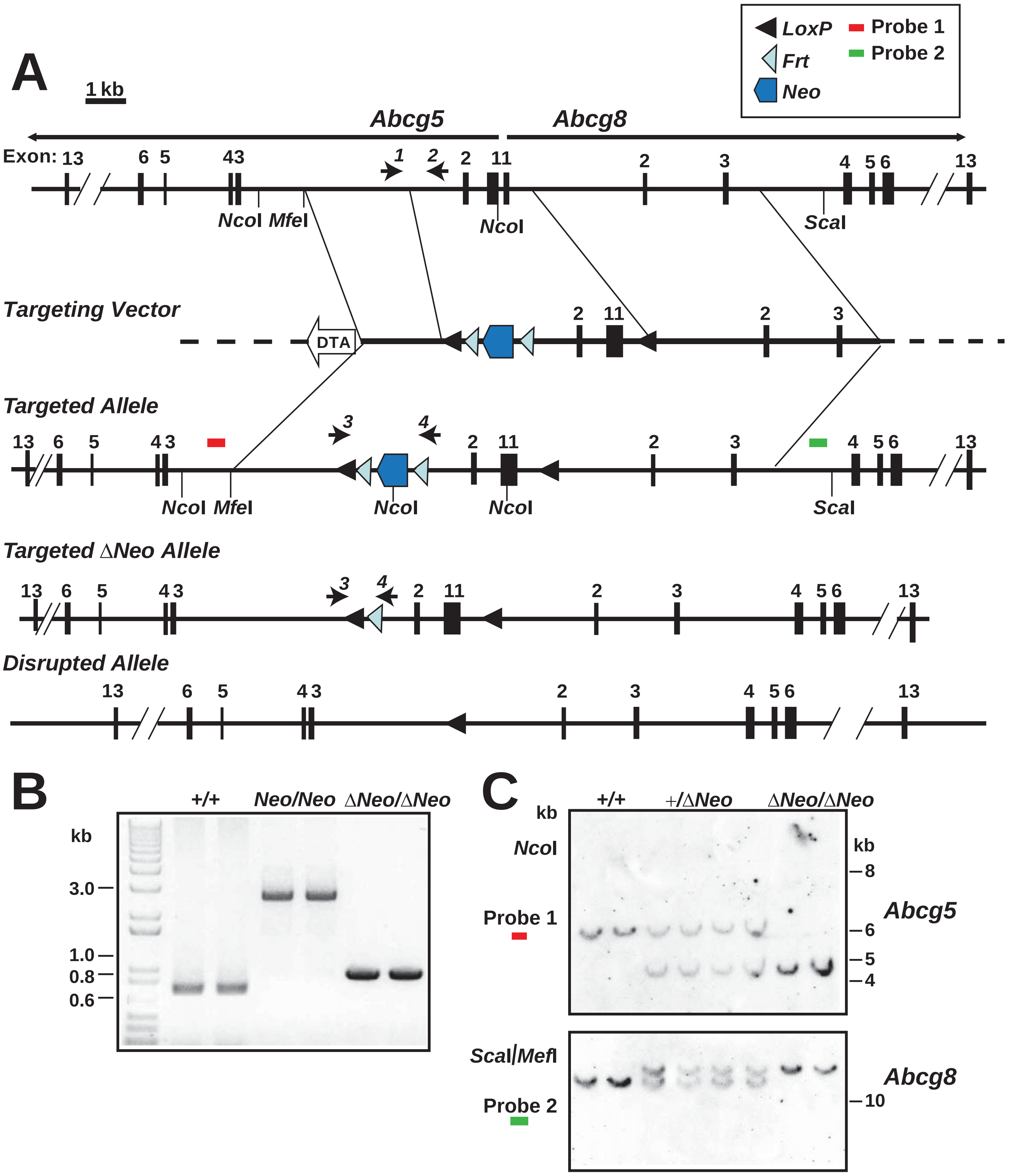
A
1 kb
LoxP
Frt
Neo
Probe 1
Probe 2
Abcg5
Abcg8
Exon: 13 6 5 43 1 2 2 11 2 3 4 5 6 13
NcoI MfeI
NcoI
ScaI
Targeting Vector
DTA
2 11 2 3
Targeted Allele
13 6 5 4 3 3 4 2 11 2 3 4 5 6 13
NcoI MfeI NcoI NcoI ScaI
Targeted ΔNeo Allele
13 6 5 4 3 3 4 2 11 2 3 4 5 6 13
Disrupted Allele
13 6 5 4 3 2 3 4 5 6 13
B
+/+ Neo/Neo ΔNeo/ΔNeo
kb
3.0
1.0
0.8
0.6
C
+/+ +/ΔNeo ΔNeo/ΔNeo
kb
NcoI
Probe 1
kb
8
6
5
4
Abcg5
ScaI/MefI
Probe 2
10
Abcg8

Fig. S2

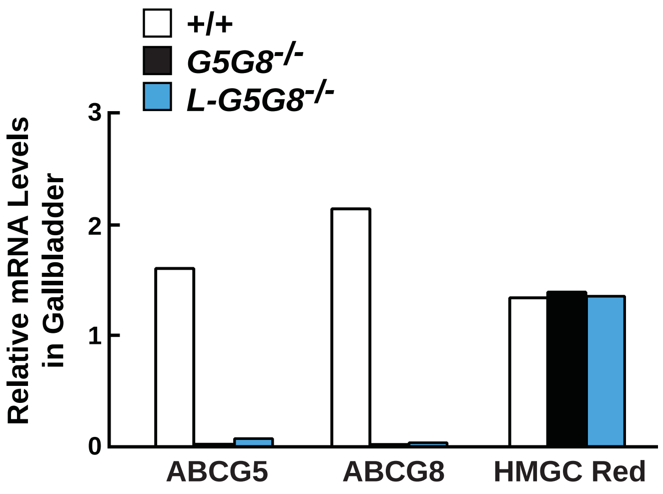
+/+
G5G8-/-
L-G5G8-/-
Relative mRNA Levels in Gallbladder
3
2
1
0
ABCG5
ABCG8
HMGC Red

Fig. S3

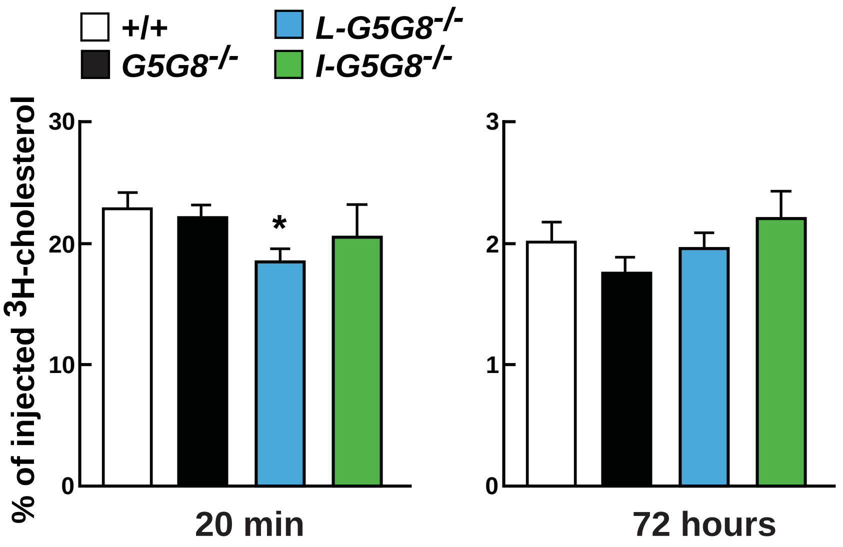

+/+
G5G8-/-
L-G5G8-/-
I-G5G8-/-
% of injected 3H-cholesterol
30
20
10
0
*
20 min
3
2
1
0
72 hours

Table SI: Oligonucleotides used for genotyping

**Construct**	**Primer**	**Sequence**
Conditional G5G8 knockout targeting allele	short arm-F	5'-GTCGACCTGATTTCCCAGCCGTGAGCC-3'
	short arm-R	5'-GTCGACGTGAGTCCCAAAGCTCTGTGACAGGAGCTTG-3'
	target arm-F	5'-CCCGGGACTCACTCTCAGCTACACCCAAC-3'
	target arm-R	5'-CCCGGGATTTATGACCTGACTTCCCAGCCG-3'
	long arm-F	5'-GTCAGCGGCCGCATAAATCCCTCTGAGGGCCACTTTCCTC-3'
	long arm-R	5'-GCTCGCGGCCGCATGGATACTTATTCAACAGTATTCGGCATC-3'
PCR assay for targeted allele	G5 intron 2-F	5'-CTCACTCCGTGGCTCTTCTGCCC-3'
	Neo R	5'-GGACGAAGAGCATCAGGGGCTCG-3'
	long arm-F	5'-GGTCATAAATCCCGGGCGAGC-3'
	G8 intron 3-R	5'-GCTAAGCCCGAACCTGGGCGTTAG-3'
Genomic blotting	probe 1 (275 bp)	F: 5'-CCTGGTGATTCCTGC-3'<br>R: 5'-CTTGTTCCCCTTTCTG-3'
	probe 2 (250 bp)	F: 5'-GGGGATTATATATGAAG-3'<br>R: 5'- CTGTGGATGTGAACC-3'
PCR assay to remove *Neo*	Primer-F	5'-CCACGGTAGGTGTCATTGTG- 3'
	Primer-R	5'- GAGCACACCAAGCAC- 3'
Genotyping	L- *G5G8*$^{-/-}$<br>I- *G5G8*$^{-/-}$	5'-GCG GTC TGG CAG TAA AAA CTA TC (for transgene forward)<br>5'-GTG AAA CAG CAT TGC TGT CAC TT (for transgene reverse)

		5'-CTA GGC CAC AGA ATT GAA AGA TCT (positive control F)<br>5'-GTA GGT GGA AAT TCT AGC ATC ATC C (positive control R)
	Wild-type and Total *G5G8*$^{-/-}$	F: 5'-CCACGGTAGGTGTCATTGTG-3'
		R1: 5'- GAGCACACCAAGCAC-3'
		R2: 5'-GGATTTGAAGCAGAAGGCAG-3'

All oligonucleotide primers are listed in pairs (F: forward, R: reverse). The tissue-specific *G5G8*$^{-/-}$ mice can be genotyped together but the wild-type and total *G5G8*$^{-/-}$ mice are genotyped separately with F+R1, and F+R2, respectively, since the PCR products are of similar size.
